# Reappraisal of the mechanism of cardiovascular responses to sympathomimetic amines in anaesthetised rats: dual α_1_-adrenoceptor and trace amine receptor mechanisms

**DOI:** 10.1007/s00210-024-03218-0

**Published:** 2024-09-06

**Authors:** Kenneth J. Broadley

**Affiliations:** Division of Pharmacology, Cardiff School of Pharmacy & Pharmaceutical Sciences, Cardiff University, King Edward Vll Avenue, Cathays Park, Cardiff, Wales CF10 3NB UK

**Keywords:** Trace amines, Infusions, Bolus dosing, Rat blood pressure, α-Adrenoceptor agonists

## Abstract

Established dogma is that sympathomimetic amines, including β-phenylethylamine (PEA), increase blood pressure by releasing noradrenaline from sympathetic neurons. Recent evidence allowing longer contact with isolated immersed tissues indicates other mechanisms. The present study re-evaluates the mechanism of pressor responses to PEA in anaesthetised rats with longer exposure to infusions. Blood pressure and heart rate were monitored by cannulating a common carotid artery of anaesthetised male Sprague–Dawley rats. Drugs were administered by bolus doses or by 20-min infusions via a cannulated jugular vein. Increases in blood pressure by bolus doses of the α-adrenoceptor agonist, phenylephrine, were converted to depressor responses by prazosin and therefore α-adrenoceptor-mediated. Pressor responses to bolus doses of PEA were reduced. PEA infusions yielded four-phase responses: An initial increase in pressure (phase 1) blocked by prazosin was due to α-adrenoceptor vasoconstriction and a secondary fall in pressure (phase 2) due to vasodilatation by nitric oxide release. A later pressure increase (phase 3), further elevated after infusion stopped (phase 4), was not attenuated by prazosin and therefore non-adrenergic. This study showed for the first time that the sympathomimetic amine, β-phenylethylamine, increases blood pressure by two mechanisms. The established indirect sympathomimetic mechanism applies to bolus dose administration. However, with prolonged exposure to infusions, an additional slow-onset sustained non-adrenergic blood pressure increase occurs, most likely mediated via trace amine-associated receptors (TAAR-1). This response will dominate with prolonged exposures in clinical practice. These results prompt a re-evaluation of established dogma on the indirect sympathomimetic mechanisms of these amines.

## Introduction

Amines including β-phenylethylamine (PEA), tyramine, amphetamine, and ephedrine have long been regarded as sympathomimetic amines because they mimic the effects of sympathetic nerve stimulation (Barger and Dale 1910). These amines exert increases in heart rate and blood pressure. They have been classified as indirectly acting sympathomimetic amines on the grounds that they release noradrenaline from neuronal vesicular storage sites (Burn and Rand 1958). However, more recent evidence suggests that this may not entirely explain the cardiovascular effects of these amines. The vasoconstrictor responses to PEA of rat (Broadley et al. 2013; Fehler et al. 2010) and guinea-pig (Broadley and Broadley 2018,2019) isolated aorta and of pig coronary arteries (Herbert et al. 2008) are not inhibited by the α_1_-adrenoceptor antagonist, prazosin. On this evidence, it has been proposed that vasoconstriction by PEA was due to stimulation of a separate class of receptors, the trace amine-associated receptors (TAARs) (Borowsky et al. 2001), so called because these amines are also known as trace amines. In transfected cell lines, TAAR-1 is a G protein-coupled receptor linked to adenylate cyclase and cAMP production (Borowsky et al. 2001). An intracellular location has also been described which is also G protein-coupled (Underhill et al. 2021). TAAR-1 receptor protein was identified by Western blotting and TAAR-1 receptor mRNA by RT-PCR in rat aorta (Fehler et al. 2010). In vivo, the vasoconstriction by these amines is seen as increases in blood pressure after intravenous administration of PEA or tyramine in rats (Day 1967; Liles et al. 2006; Khwanchuea et al. 2008), cats (Day 1967; Burn and Rand 1958), dogs (Kohli and Goldberg 1982; Woodman and Pannangpetch 1994), and rabbits (Du et al. 1992). In humans, the administration of tyramine (Peatfield et al. 1983; Colombo et al. 1989) and phenylpropanolamine (Salerno et al. 2005) also increase blood pressure. There is no information on whether TAARs are involved in the in vivo cardiovascular effects of trace amines. Most of the evidence indicates that increases in blood pressure are due to indirect sympathomimetic mechanisms. For example, the pressor actions of tyramine are blocked by the depletion of noradrenaline stores with reserpine (Burn and Rand 1958).

Thus, in vivo findings suggest that tyramine, β-PEA, and related amines behave as indirectly acting sympathomimetic amines, whereas in vitro observations in isolated blood vessels suggest the involvement of TAAR receptors. A possible reason for this discrepancy is that most in vitro studies have involved immersed tissues allowing prolonged contact with the amines. In contrast, in vivo clinical studies (Bianchetti et al. 1982; Peatfield et al. 1983; Meck et al. 2003) have usually examined bolus dosing in which there is only a brief peak concentration of agonist. The rate of onset of TAAR-mediated vasoconstriction by PEA in guinea-pig aorta is significantly slower, peaking at 13.6 min, than for the α-adrenoceptor agonist, phenylephrine, which peaked at 3.9 min (Broadley and Broadley 2019). Therefore, in in vivo studies, bolus dosing may have prevented observation of the delayed and slower TAAR-1-mediated responses to these amines. The clinical use of the vasoconstrictor activity of sympathomimetic amines in raising blood pressure is to treat the severe hypotension associated with septic and anaphylactic shock. Phenylephrine and ephedrine have been used in septic shock (Chen and Wong 2022), and phenylephrine has been used in anaphylactic shock (Luhmann et al. 2013), by bolus dosing. There is limited information on infusions of these amines, but phenylephrine infusions have been employed to maintain blood pressure during spinal anaesthesia for caesarean section (Ngan Kee et al. 2004). There is no information on the use of PEA in septic shock, anaphylaxis or spinal anaesthesia. The present study was therefore undertaken to examine the in vivo responses to PEA on anaesthetised rat blood pressure and heart rate when administered by bolus dosing and by infusion that allows the slow-onset responses to develop fully.

## Methods

### Anaesthetised rats

Male Sprague–Dawley rats (236–423 g; 8 and 12 weeks old) were obtained from Charles River (Harlow, UK). Rats were maintained in conventional animal housing, subjected to a 12-h light–dark cycle (8am–8pm) at a room temperature of 21 °C and humidity of 55 g.m^3^. This study was undertaken only in male rats. It is acknowledged that the results may differ in females; however, it was decided in the first instance to avoid any influences of variations in female sex hormones during the oestrus cycle and the necessity of having to determine the stage of the cycle in each rat prior to each experiment.

Rats were anaesthetised with 60 mg/kg i.p. pentobarbitone sodium (Khwanchuea et al. 2008). The rat was placed on a Harvard heated table maintaining the rat’s temperature at 37 °C by a Harvard (Edenbridge, Kent, UK) homeothermic table control unit. An intravenous polythene cannula was inserted into a jugular vein and heparin administered intravenously (100 units/100 g). A common carotid artery was closed with an artery clip before cannulating with a tapered polythene cannula filled with heparinised saline (100 units/ml). The arterial cannula was attached to a PDCR 75 S/N 18131 pressure transducer (Druck Ltd, Leicester, UK) in series with a Condon manometer. The pressure in the transducer and manometer was raised to 100 mmHg before releasing the artery clip. Arterial blood pressure was monitored using a Powerlab 4SP data acquisition system (ADInstruments, Chalgrove, Oxfordshire, UK) and the pulse signal used as a trigger for heart rate.

### Experimental protocol

Drugs were administered intravenously by inserting a hypodermic needle into the cannula, injecting the required volume of drug made up in saline, pinching the tube to remove the needle and inserting a second needle attached to a syringe filled with saline. All drugs were washed in immediately with 0.2 ml of saline. Alternatively, PEA was administered by infusion for 20 min via the intravenous cannula. An Imed 800 (Pharmacia, Milton Keynes, UK) slow infusor fitted with a 10-ml syringe was used. At the completion of the infusion, the cannula was flushed through with 0.2 ml saline. All doses are expressed as mg or µg base/100 g body weight (BW) for bolus dosing or mg base/100 g body weight (BW)/min for infusions and hereafter are given as mg/100 g or mg/100 g/min.

### Data processing and statistical analysis

Systolic and diastolic arterial blood pressures (mmHg) and heart rate (beat/min) were recorded. With bolus doses, the changes in blood pressure and heart rate at the peak effect were measured and the difference from the pre-dose baseline was recorded. With infusions, the blood pressures and heart rate were measured at minute intervals for 5 min and then every 5 min. After infusions were stopped, blood pressure and heart rate were continued to be measured initially at minute intervals for 5 min and then at 5-min intervals for a further 10 min. For bolus dose administration, changes in diastolic and systolic blood pressure and heart rate from baseline were calculated and plotted as the mean ± SEM. For infusions, time courses of the responses were plotted as the mean ± SEM absolute diastolic and systolic blood pressures or heart rate against time. Mean responses obtained before and after antagonism by prazosin or before and after infusions of PEA were compared by Student’s paired *t*-tests using Microsoft Excel. Differences were considered significant when *P* ≤ 0.05. Blinding was not necessary because no comparisons were made between test and control animals. Comparisons of blood pressure and heart rate changes were usually made within the same animal.

## Materials

Drugs used were heparin sodium salt from porcine intestinal mucosa (180 units/mg), prazosin hydrochloride, (-)-phenylephrine hydrochloride, and β-phenylethylamine hydrochloride (PEA) obtained from Sigma-Aldrich (Poole, Dorset, UK) and sodium pentobarbitone (200 mg/ml) from Merial, Woking UK). PEA and phenylephrine were dissolved in distilled water. Prazosin hydrochloride was dissolved (1 mg/ml) in dimethylsulfoxide (DMSO)/normal saline (50:50). Other drugs were dissolved in normal saline.

## Results

### Bolus doses of phenylephrine and PEA

**Fig. 1:**
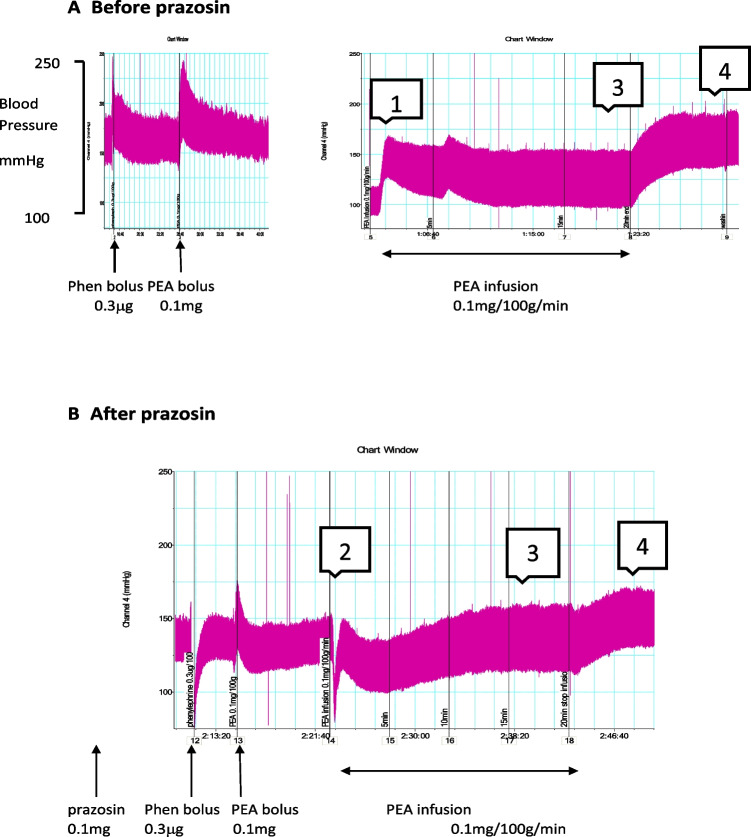
Typical trace of the blood pressure responses of an anaesthetised rat to bolus doses of phenylephrine (Phen, 0.3 µg/100 g body weight) and β-phenylethylamine (PEA, 0.1 mg/100 g body weight) and to an infusion of PEA (0.1 mg/100 g/min) for 20 min. Administration was made before (**A**) and repeated after slow intravenous dosing with prazosin (0.1 mg/100 g) (**B**). The responses to PEA infusions were divided into four phases. These are an initial increase in pressure (phase 1) seen only before prazosin. This was followed by a fall in pressure (phase 2), which was most pronounced in the presence of prazosin. A secondary increase in pressure (phase 3) occurred, while the infusion continued and after stopping the infusion this increases further (phase 4)

**Fig. 2:**
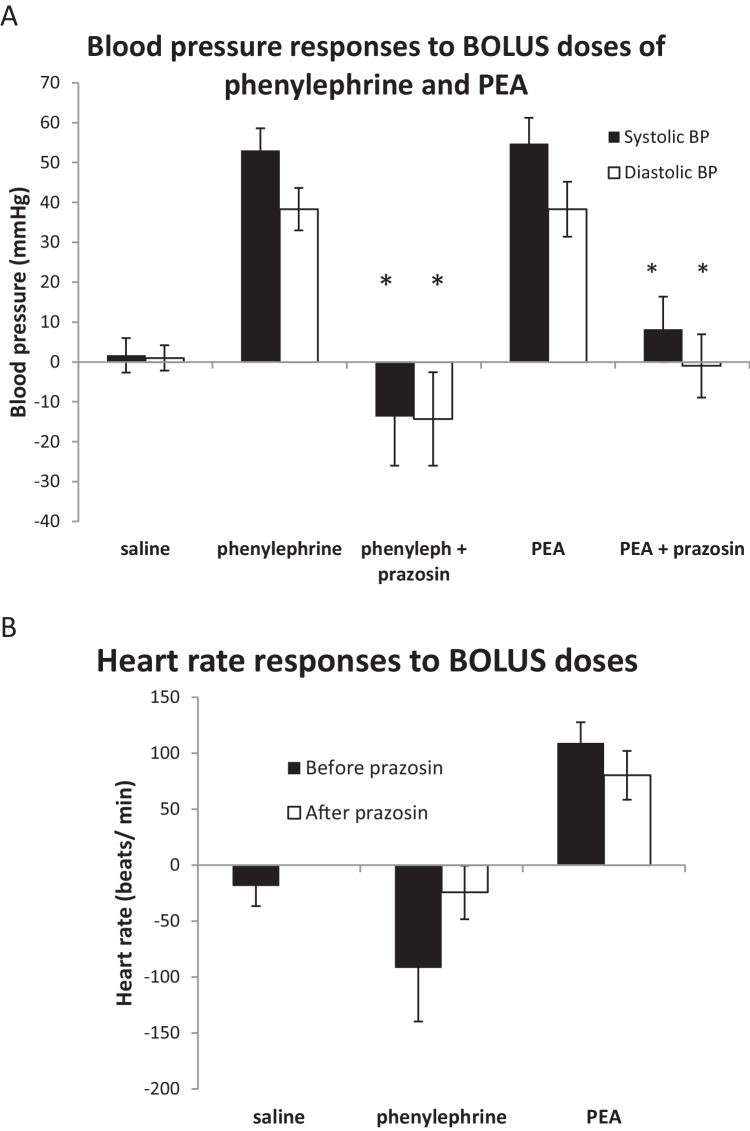
Mean (*n* = 6) blood pressure (**A**) and heart rate (**B**) responses of anaesthetised rats to intravenous bolus doses of saline (0.1 ml/100 g body weight), phenylephrine (0.3 µg/100 g body weight), and β-phenylethylamine (PEA, 0.1 mg/100 g body weight) before and after prazosin (0.1 mg/100 g). Changes in systolic (solid bars) and diastolic (open bars) blood pressures are shown together with SEM. Heart rate changes are shown before (solid bar) and after prazosin (open bar). Asterisk denotes a significant difference between before and after prazosin (*P* < 0.05) by Student’s paired *t*-test

Bolus dosing of the α-adrenoceptor agonist, (-)-phenylephrine hydrochloride (0.3 μg/100 g), increased systolic and diastolic blood pressure by 53.0 ± 5.6 and 38.3 ± 5.3 mmHg (*n* = 6), respectively (Figs. 1A and 2A). Heart rate was reduced by 91.7 beats min^−1^ (bpm) (Fig. 2B). Bolus doses of PEA (0.1 mg/100g) increased systolic and diastolic blood pressure by 54.7 ± 6.5 and 38.3 ± 6.9 mmHg (Figs. 1A and 2A) and increased heart rate by 109.2 ± 18.6 beats/min (Fig. 2B). These doses were selected from preliminary experiments to be submaximal.

### Intravenous infusions of PEA

**Fig. 3:**
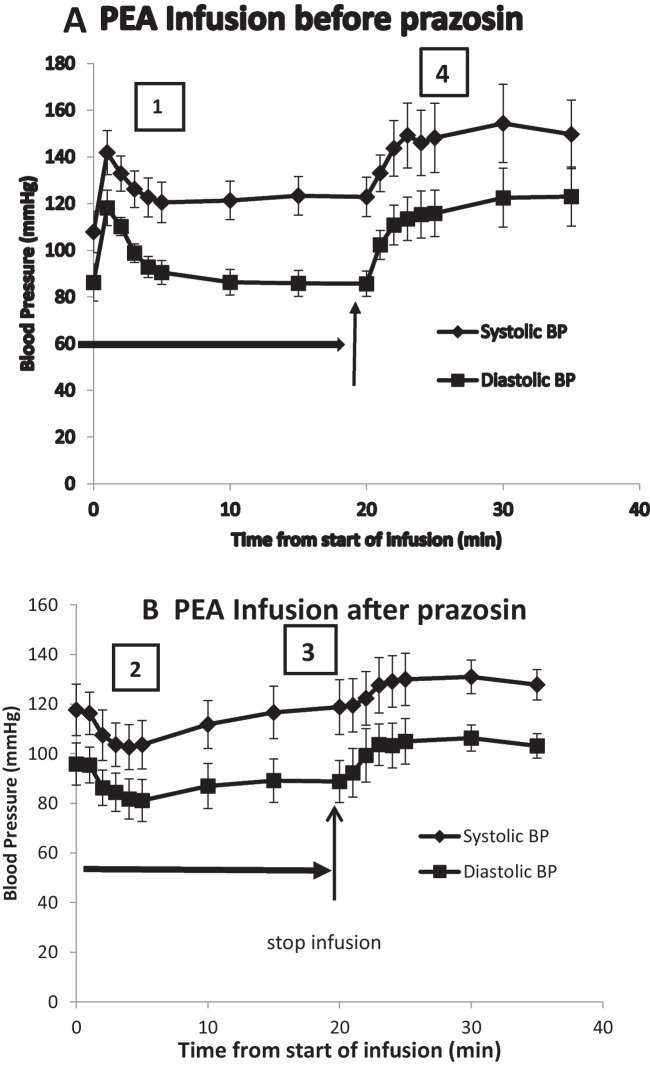
Mean (*n* = 7) systolic (black diamond) and diastolic (black square) blood pressures of anaesthetised rats during and immediately after 20-min intravenous infusions of β-phenylethylamine (PEA, 0.1 mg/100 g body weight), shown by the horizontal arrow. Infusions were administered before (**A**) and after (**B**) prazosin (0.1 mg/100 g). The four phases of the blood pressure responses are shown: (1) the initial increase in blood pressure, (2) a secondary fall seen mainly after prazosin, (3) a secondary increase in pressure and (4) a sustained increase that continues after stopping the infusion

**Fig. 4:**
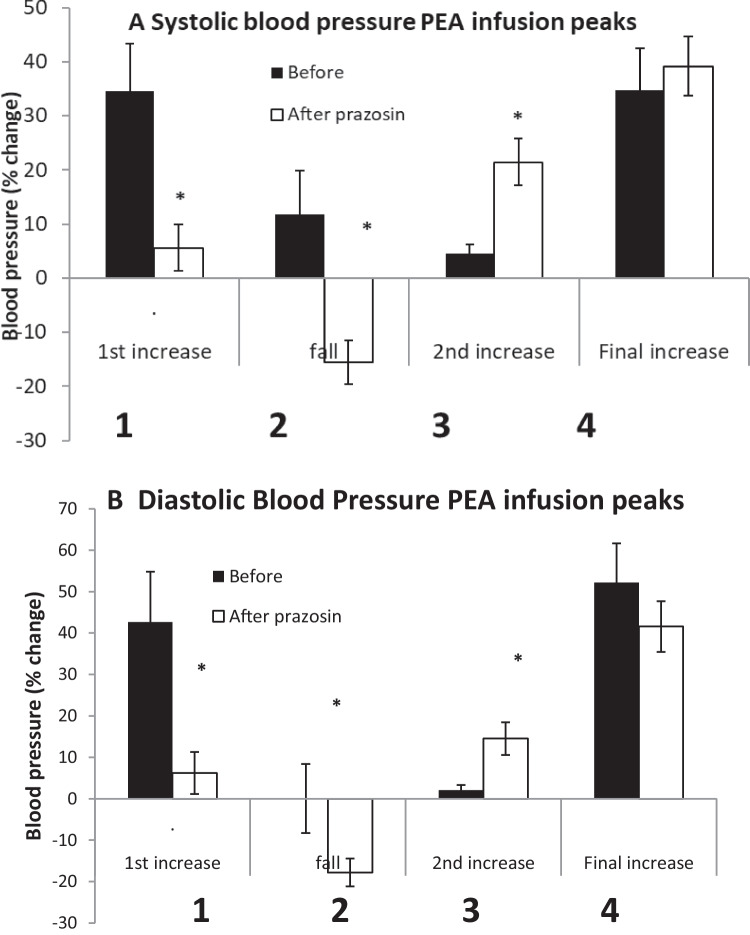
Mean (*n* = 7) peaks of the four phases (labelled 1–4) of the **A** systolic and **B** diastolic blood pressure changes during infusion of β-phenylethylamine (PEA, 0.1 mg/100 g body weight) for 20 min to anaesthetised rats. PEA infusions were made before and after intravenous prazosin (0.1 mg/100 g). The four phases shown in Fig. 1 are as follows: (1) the initial increase in blood pressure, (2) a secondary fall seen mainly after prazosin, (3) a secondary increase in pressure and (4) a sustained increase that continues after stopping the infusion. Peak responses are measured as the change in blood pressure from the pre-infusion baseline (phases 1 and 2) or as the change from the secondary fall in the case of phases 3 and 4, expressed as a percentage. Asterisk denotes a significant difference between before and after prazosin (*P* < 0.05) by Student’s paired *t*-test

A 20-min intravenous infusion of PEA (0.1 mg/100 g/min) produced a four-phased blood pressure response (Figs. 1A and 3). An initial increase (Peak 1) peaked at 1.3 ± 0.2 min, systolic and diastolic blood pressure increasing by 35.0 ± 7.2 and 33.5 ± 6.7 mmHg, respectively (34.5 ± 8.8 and 42.6 ± 12.2%) (Fig. 4). Blood pressure then returned towards baseline, this fall peaking at 8.2 ± 2.4 min, systolic BP decreasing to 10.7 ± 8.1 mmHg (11.8 ± 8.1%) above baseline and diastolic to 2.0 ± 2.8 mmHg (0.04 ± 8.3%) below baseline (Peak 2). A secondary increase (Peak 3) in blood pressure occurred while the infusion continued, which peaked at 17.5 ± 1.2min, systolic and diastolic pressures increasing by 7.1 ± 2.2 (4.6 ± 1.7%) and 1.7 ± 1.1 mmHg (2.0 ± 1.3%) above the Peak 2 fall in pressure. After the infusion was stopped at 20 min, there was a further increase in pressure (Peak 4) peaking at 28.5 ± 2.2 min, systolic and diastolic increasing by 41.2 ± 9.6 (34.7 ± 7.8%) and 43.5 ± 7.6 mmHg (52.2 ± 9.5%) above the phase 2 fall in pressure.

**Fig. 5:**
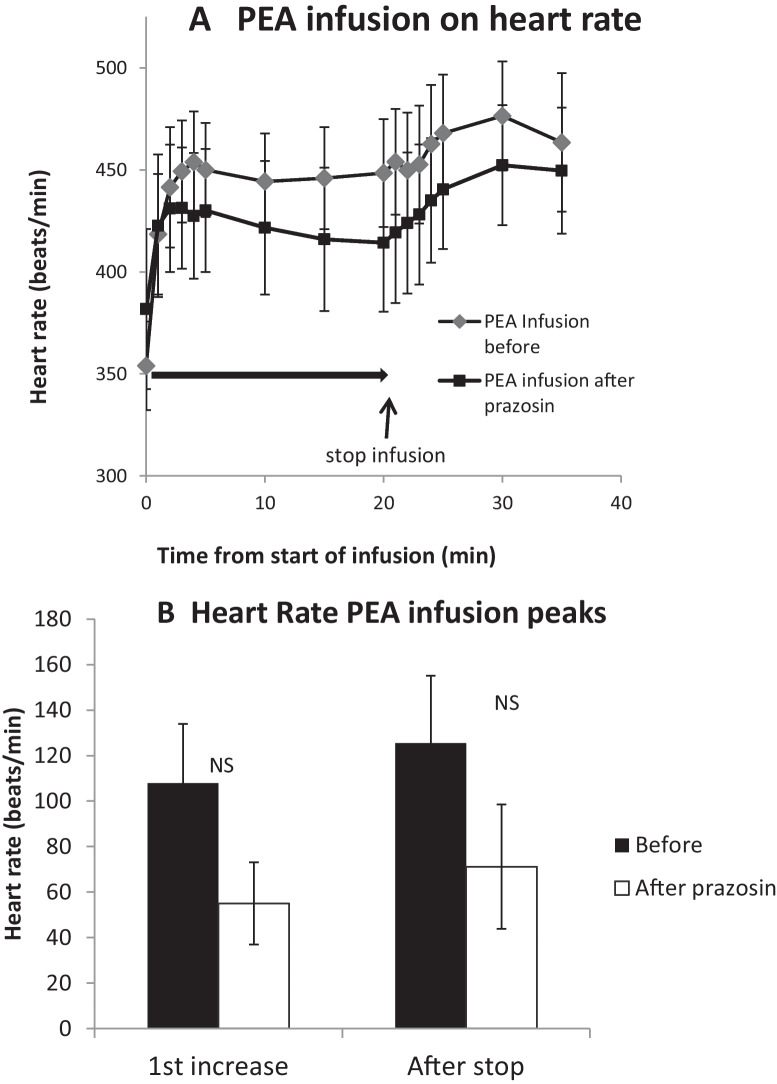
Mean (*n* = 7) peak increases in heart rate during (first increase) and after stopping an infusion of β-phenylethylamine (PEA, 0.1 mg/100 g body weight) for 20 min to anaesthetised rats. PEA infusions were made before and after intravenous prazosin (0.1 mg/100 g). **A** Mean time courses before (black diamond) and after (black square) prazosin. **B** Mean peak increases in heart rate before (solid bar) and after (open bar) prazosin. Peak responses are measured as the change in heart rate (beats/min) from the pre-infusion baseline

Heart rate increased by 99.8 ± 22.4 beats/min at 7.2 ± 3.1 min into the infusion of PEA. This was followed, after stopping the infusion, by a secondary increase which peaked at 122.5 ± 29.4 beats/min above baseline at 28.2 ± 1.9 min (Fig. 5).

### Effects of the α_1_-adrenoceptor antagonist prazosin

The α_1_-adrenoceptor antagonist, prazosin (0.1 mg/100 g slowly), converted the pressor responses to bolus doses of phenylephrine to a depressor response (systolic − 13.7 ± 12.3; diastolic − 14.3 ± 11.7 mmHg) (Figs. 1B and 2A). The pressor responses to bolus doses of PEA were significantly inhibited by prazosin but not abolished (systolic 8.2 ± 8.2; diastolic − 1.0 ± 7.9 mmHg) (Figs. 1B and 2A). The increases in heart rate by bolus doses of PEA (80.4 ± 21.8 beats/min) were not affected (Fig. 2B).

After prazosin, the PEA infusion showed a three-phase pressure response (Figs. 1B and 3B). The initial increase (Peak 1) was abolished and replaced by an immediate fall in pressure below baseline (Peak 2) (systolic − 19.3 ± 5.7 mmHg (− 15.6 ± 4.0%), diastolic − 14.7 ± 2.7 mmHg (− 17.8 ± 3.4%)) (Fig. 4). The secondary increase in pressure (Peak 3) was significantly greater than before prazosin (systolic 21.3 ± 5.1 (21.5 ± 4.3%), diastolic 8.2 ± 1.6 mmHg (14.5 ± 4.0%) above the fall) (Fig. 4). The final increase in blood pressure (Peak 4) that occurred after ceasing the PEA infusion (systolic 38.0 ± 5.2 mmHg (39.2 ± 5.6%), diastolic 25.3 ± 3.9 mmHg (41.6 ± 6.1%) above the fall) was not significantly different from before prazosin (Fig. 4).

The heart rate response to PEA infusion was not affected by prazosin (Fig. 5). It was still biphasic, with an initial increase peaking at 49.5 ± 18.2 beats/min and a secondary increase occurring after the infusion was completed (70.5 ± 27.7 beats/min). Neither phase was significantly different from before prazosin (Fig. 5B).

### Effect of PEA infusion on bolus dose responses to phenylephrine and PEA

**Fig. 6:**
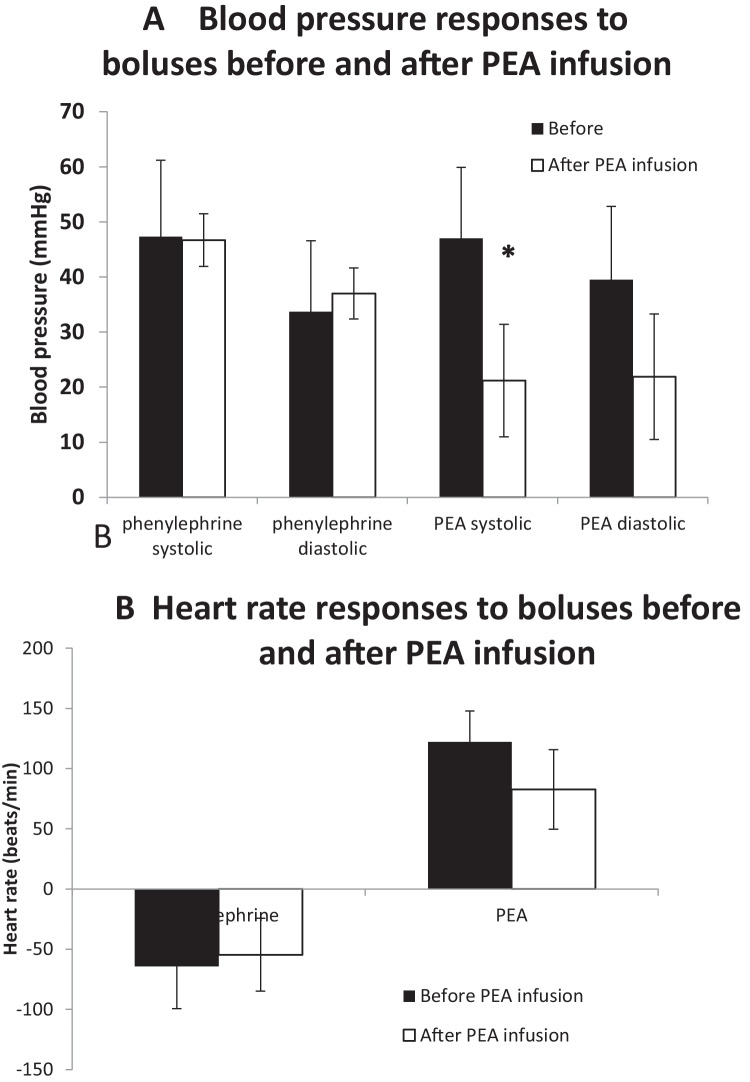
Mean (*n* = 4) peak systolic and diastolic blood pressures (**A**) and heart rate (**B**) responses to bolus doses of phenylephrine and β-phenylethylamine (PEA) to anaesthetised rats. Responses were obtained before (solid bar) and after (open bar) an infusion of β-phenylethylamine (PEA, 0.1 mg/100 g body weight) for 20 min. Asterisk denotes a significant difference between before and after PEA infusion (*P* < 0.05) by Student’s paired *t*-test

The increases in blood pressure in response to bolus doses of phenylephrine administered before and after a 20-min infusion of PEA (0.1 mg/100 g/min) were not significantly different (Fig. 6A). The blood pressure increases to bolus doses of PEA were, however, reduced after the PEA infusion. The increase in systolic blood pressure was significantly reduced from 47.0 ± 12.9 to 21.2 ± 10.2 mmHg, although the change in diastolic blood pressure was not significant (Fig. 6A). The heart rate responses to phenylephrine and PEA bolus doses were not affected by the PEA infusion (Fig. 6B).

### Effects of saline infusions on blood pressure and heart rate

**Fig. 7:**
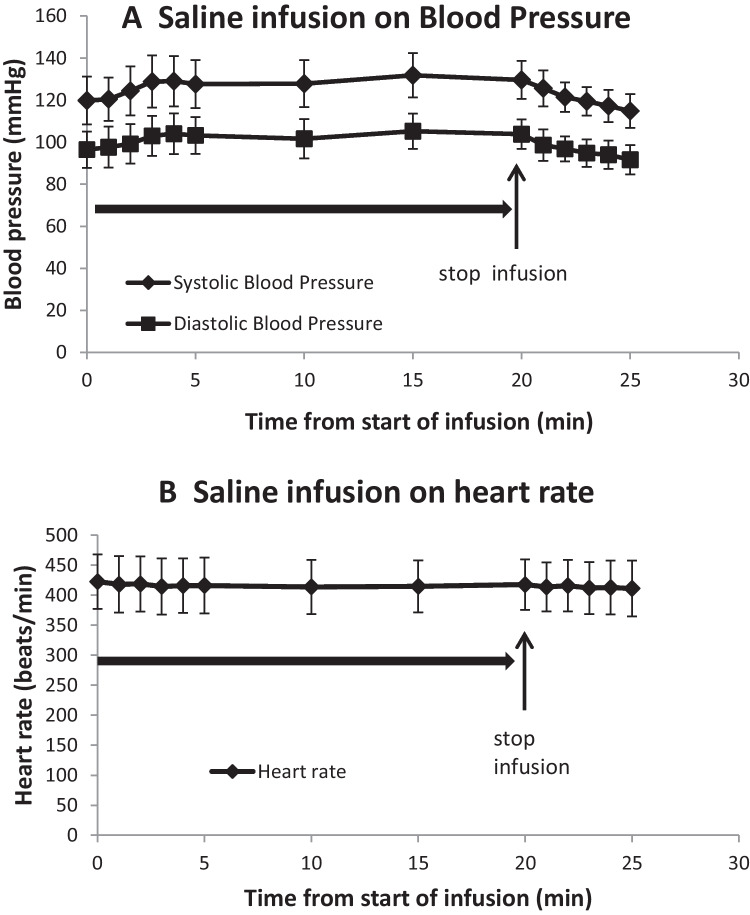
Blood pressure and heart rate responses of anaesthetised rats to saline infusion. Mean (*n* = 5) systolic (black diamond) and diastolic (black square) blood pressures (**A**) and heart rates (**B**) of anaesthetised rats during (horizontal arrow) and after infusions of saline (0.1 ml/100g/min) for 20 min

Infusion of saline (0.1 ml/100 g/min) for 20 min produced a slight non-significant increase in blood pressure, systolic increasing from 119.8 ± 11.3 to a peak of 131.8 ± 10.5 mmHg and diastolic increasing from 96.4 ± 8.6 to 105.2 ± 8.4 mmHg at 15 min into the infusion (Fig. 7A). On completing the infusion at 20 min, blood pressure immediately fell, systolic and diastolic pressures reaching 114.8 ± 8.0 and 91.6 ± 7.0 mmHg, respectively. Heart rate showed a slight fall over the course of the saline infusion from 422.6 ± 45.5 to 413.8 ± 45.3 beats/min at 10 min into the infusion and 411.2 ± 46.6 beats/min at 25 min (Fig. 7B).

## Discussion

Biogenic amines such as β-phenylethylamine (PEA), tyramine, ephedrine and amphetamine increase blood pressure by vasoconstriction, which is classically regarded as being through an indirect sympathomimetic action. That is, they release noradrenaline from sympathetic nerve endings onto vascular smooth muscle α-adrenoceptors (Burn and Rand 1958). The present study confirms this well-established mechanism for a bolus dose of PEA, since the selective α_1_-adrenoceptor antagonist, prazosin, significantly reduced the pressor response to a bolus dose of PEA. The reference α-adrenoceptor agonist, phenylephrine, also exerted increases in blood pressure due to direct α_1_-adrenoceptor-mediated vasoconstriction which was also abolished by prazosin and converted to a depressor response. The vasodepressor response to phenylephrine after α_1_-adrenoceptor blockade could be attributed to β-adrenoceptor-mediated vasodilatation. A residual pressor response to bolus dosing with PEA after prazosin blockade was therefore not mediated via α_1_-adrenoceptors.

The blood pressure response to intravenous infusions of PEA was more complex than with bolus dosing. The response could be divided into four phases. Phase 1 was an initial increase in blood pressure which was short-lived, peaking at 1.3 min into the infusion. This increase in blood pressure was abolished by the α_1_-adrenoceptor antagonist, prazosin, indicating that it was mediated via vascular α_1_-adrenoceptors and equivalent to the pressor response seen with bolus dosing.

In the presence of α_1_-adrenoceptor blockade, the first component of the response to PEA infusion was an immediate fall in blood pressure (Phase 2). This component was less evident in the absence of α_1_-adrenoceptor blockade. Vasodilatation was the sole response to PEA and tyramine in rat isolated mesenteric vascular beds (Broadley et al. 2009; Anwar et al. 2012). This vasodilatation was abolished by the nitric oxide synthase (NOS) inhibitor N_ω_-nitro-L-arginine methyl ester (L-NAME) indicating that it was mediated via the release of nitric oxide (NO), probably from the vascular endothelium. However, the vasodilator responses to tyramine and PEA in rat isolated aorta were found to be endothelium-independent (Varma and Chemtob 1993). The vasodilator component of the response to infusions of PEA in the present study was therefore likely due to NO release. Tyramine infusions in humans also increased forearm blood flow indicating a vasodilator response (Jacob et al. 2003). Further evidence for the vasodilator activity of these amines was the potentiation of their vasoconstrictor actions by inhibition of NOS with L-NAME in guinea pig aortic rings (Broadley and Broadley 2018) and of their pressor responses in conscious rabbits (Du et al. 1992). Other mechanisms to explain this vasodilator component such as β-adrenoceptors, 5-HT-, histamine- and adenosine receptors or cyclooxygenase products have been excluded (Varma and Chemtob 1993). It is likely that a vasodilator response to these amines occurs predominantly in certain vascular beds such as the mesenteric circulation, whereas vasoconstriction predominates elsewhere.

Following the vasodilator response to PEA infusion was a slow recovery of the blood pressure while the infusion continued (phase 3). When the infusion was stopped, this increase in blood pressure continued, reaching a level greater than the initial pressor response (phase 4). These latter two phases appeared to be continuous and were not inhibited by prazosin. It is possible that the secondary rise in blood pressure could be due to a rebound reflex vasoconstriction. It is possible that during the infusion, there is activation of baroreceptors, for example, in the right atrium, which induces a reflex vasodilatation. When the infusion stops, there may then be a compensatory vasoconstriction. This possibility was tested by making a 20-min saline infusion of the same rate. No rebound increase in blood pressure occurred on stopping the infusion. The secondary increase in blood pressure on stopping the PEA infusion was therefore a persistent effect of the PEA. The precise reason for the secondary increase in pressure on stopping the infusion is unclear. One possibility is that the opposing vasodilatation occurring during the infusion is rapidly reversed on stopping the infusion, but the slow-onset vasoconstriction continues to develop and persists beyond ceasing the infusion. Because both phase 3 and 4 pressor effects were not blocked by prazosin, it could be concluded that they were not due to α_1_-adrenoceptor stimulation. There are no reports in the literature of this delayed pressor response to PEA or other trace amines. However, in unpublished data, an identical multiphasic pressor response was demonstrated with infusions of dexamphetamine. A similar non-adrenergic vasoconstriction to PEA has been observed in isolated aortae from rats (Broadley et al. 2013; Fehler et al. 2010) and guinea pigs (Broadley and Broadley 2018, 2019), in pig coronary arteries (Herbert et al. 2008; Koh et al. 2019) and in human mammary artery and saphenous vein (Broadley and Mehta 2023). A non-adrenergic coronary vasoconstriction was also observed for S-(-)-cathinone in guinea-pig isolated perfused hearts (Al-Motarreb and Broadley 2004). This non-adrenergic vasoconstriction was shown to be slow in onset and persistent in guinea-pig aorta (Broadley and Broadley 2019), perfused hearts (Al-Motarreb and Broadley 2004) and human saphenous vein and mammary artery (Broadley and Mehta 2023), which contrasts with the rapid onset and non-sustained contractions to the α-adrenoceptor agonist phenylephrine. Furthermore, octopamine causes a fast-onset contraction in guinea-pig aorta mediated via α_1_-adrenoceptors and a slow-onset contraction when α_1_-adrenoceptors are blocked (Broadley et al. 2013).

**Fig. 8:**
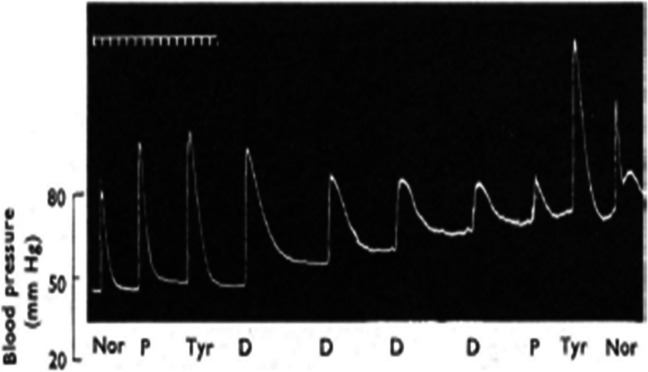
Pressor responses of a pithed rat (250 g) to bolus doses of noradrenaline (Nor, 50 ng), β-phenylethylamine (P, 25 μg), tyramine (Tyr, 25 μg), and dexamphetamine (D 25 μg). Tachyphylaxis to the pressor responses was established by repeated administration of dexamphetamine, after which there was cross-tachyphylaxis to β-phenylethylamine but not to tyramine, and no change in the response to noradrenaline. During the repeated dosing with dexamphetamine, the resting blood pressure did not return to baseline (reproduced with permission from Day (1967))

Further evidence that the mechanism of vasoconstriction by the trace amine PEA differs from that of phenylephrine came from bolus dose administration after the PEA infusion. When the bolus dose of phenylephrine was repeated after PEA infusion, the responses were not reduced. However, when the PEA bolus was repeated, the pressor response was reduced but not abolished. This indicates a degree of tachyphylaxis to the response to bolus dosing. Tachyphylaxis is a common phenomenon with indirectly acting sympathomimetic amines, whereby repeated administration of bolus doses results in progressively smaller responses (Day 1967). This establishes that bolus doses of PEA increase blood pressure in part by an indirect sympathomimetic action through the release of neuronal noradrenaline. Inspection of the blood pressure trace in the original paper by Day (1967) shows a gradual increase in basal blood pressure of a pithed rat as bolus doses with amphetamine were repeated (Fig. 8). This phenomenon was not referred to. However, a likely explanation that can be proposed from the present study is that the slow-onset and persistent non-adrenergic component of the vasoconstriction from each dose accumulates and leads to the observed rise in resting blood pressure.

It is clear therefore that bolus doses of PEA cause a rapid onset but transient vasoconstriction that is mediated predominantly by an indirectly acting sympathomimetic action via α_1_-adrenoceptors. In contrast, infusion of PEA causes a brief sympathomimetic response, a short-lived fall in blood pressure followed by a slow onset but persistent vasoconstriction that is non-adrenergic and outlives the infusion. The question is, what is the mechanism of this latter vasoconstriction? A primary candidate for mediating this response is the trace amine-associated receptor-1 (TAAR-1). TAAR-1 has been detected from mRNA by RT-PCR in rat heart (Chiellini et al. 2007) and from receptor protein by Western blotting and from mRNA by RT-PCR in rat aorta (Fehler et al. 2010). TAAR-1 has also been identified from mRNA in several human cardiovascular tissues including the kidney and spleen (Borowsky et al. 2001). The slow-onset persistent vasoconstriction is therefore proposed to be due to stimulation of vascular TAAR-1.

Although the vascular response to trace amines was the main focus of this study, heart rate responses were also routinely monitored. Bolus doses of the reference α-adrenoceptor agonist, phenylephrine, exerted a fall in heart rate. This could be attributed to a reflex bradycardia induced by the rise in blood pressure since it was abolished when the pressor response was blocked by prazosin. The rise in blood pressure is detected by carotid sinus baroreceptors and the reflex bradycardia mediated via vagal nerve activity (Broadley 1996). PEA in contrast increased heart rate, presumably through β-adrenoceptor stimulation due to its indirect sympathomimetic activity. PEA infusion also increased heart rate, which was not blocked by prazosin. There was a small further increase in heart rate when the infusion was stopped. This was not a rebound effect from the increased volume of the infusion since no similar increase in heart rate occurred upon stopping a saline infusion. The increases in heart rate with bolus doses of PEA were not affected after PEA infusions. This indicates that no desensitisation of the β-adrenoceptor-mediated responses to PEA had occurred. It also indicates no significant tachyphylaxis of the indirect sympathomimetic effect of PEA on the heart.

This study has shown for the first time that the sympathomimetic amine, β-phenylethylamine (PEA), exerts increases in blood pressure by two mechanisms. The established indirect sympathomimetic amine mechanism of noradrenaline release from adrenergic neurones applies to bolus dose administration. However, with more prolonged exposure as occurs with infusions, there is an additional slow onset and sustained vasoconstriction and increase in blood pressure. This is non-adrenergic and most likely mediated via TAAR-1. A minor limitation of this study is that only one trace amine, PEA, was reported here. However, preliminary studies with amphetamine have indicated similar results. A further limitation of the extension to the clinic is that the rats were anaesthetised. The delayed TAAR-1-mediated response has been largely ignored in experimental pharmacology because of the widespread use of bolus dosing. However, it has dominant significance in practice. Firstly, in the clinical use of those sympathomimetic amines which also activate TAAR-1, their oral and topical application will have the required duration of exposure for this mechanism. Secondly, dietary amines such as tyramine will have the required prolonged exposure in the gut and mesenteric circulation to facilitate digestion and absorption from the gut (Broadley et al. 2009). In the light of these novel findings, the established dogma of the indirect sympathomimetic mechanism for the cardiovascular actions of biogenic amines needs to be re-evaluated. With more clinically relevant exposure to these amines, the trace amine receptor mechanism becomes more dominant and long-lasting.

## Data Availability

Data is provided within the manuscript, and any further details may be obtained directly from the corresponding author.
